# Supplementary material for: Chromothripsis during telomere crisis is independent of NHEJ, and consistent with a replicative origin
Source: Genome Res. 2019 May;29(5):737–49. doi: 10.1101/gr.240705.118 (PMC6499312; doi:10.1101/gr.240705.118)
Supplement: Supplemental Material [file supp_gr.240705.118_Supplemental_file_1.zip › contigs/annotated_contigs/DB37/contig.2.DB37_length_315_mean_cov_17.0507936508.docx]

**DB37_length_315_mean_cov_17.0507936508**

```
GGGAGTCCTAGGATGCTTCTGGCTTCAATGCCGTTTGTCTCAATATGGGGAGGCTGCCCAACTTCAACAGCGTTACTGAAGGAGGATGG
 >chr15:91508509-91508760 - E=5e-131 p=0e+00
ACATAAACTGCACACATGTGAAGCGTAAGAGAAAAACCAGCGCCACAGTCCAGTTAACCAAGTCTCTCTACCCCAGCAGTTCTTCCCAC

CCCGTTCTCACGCCTCCTTCCCAGCCTCACCCTGCCGAGCAACCACTGATTTTTTTTTTTTTTTTTTTTTTT|T|GTTTTTTTTTTTTT
                                                                         >chrX:56328299
TTTTTGTTTTTGTG|TTGTTCAATTTTACTTCTACTTAATCTTCTCTCTTT-56328328 + E=5e-03
```
